# Assessing PD‐L1 expression in non‐small cell lung carcinoma: a prospective study of matched fine‐needle aspirates, core biopsies, and resection specimens using alcohol and forming fixatives

**DOI:** 10.1002/2056-4538.70041

**Published:** 2025-08-25

**Authors:** Alexander Haragan, Natalie Kipling, Michael Shackcloth, John R Gosney, Michael P Davies, John K Field

**Affiliations:** ^1^ Institute of Systems, Molecular and Integrative Biology University of Liverpool Liverpool UK

**Keywords:** assay, digital microscopy, immunocytochemistry, lung

## Abstract

PD‐L1 expression for the prediction of response to immune‐checkpoint blockade remains the most broadly utilised clinically validated biomarker in a range of tumour types. In this study, we aimed to assess, in a prospectively collected matched cohort, the impact of sampling technique and both formalin and alcohol fixation on PD‐L1 expression and heterogeneity in non‐small cell lung carcinoma (NSCLC). Patients undergoing surgical resection for NSCLC were consented. Surgical specimens were received directly from theatre and sampled fresh to produce two sets of core biopsies, two fine‐needle aspirates (FNAs) and two whole‐block tissue sections from each specimen. A matched biopsy, FNA, and whole‐block were placed into formalin or an alcohol‐based fixative (Cytolyt™) prior to PD‐L1 immunohistochemistry assessment. A total of 114 specimens from 57 patients were included. All whole‐block cases (100%), 92% of core biopsies, and 88% of FNAs were adequate for PD‐L1 expression analysis. Fixation had no significant impact on adequacy, but cytology specimens fixed in alcohol showed a significant reduction in PD‐L1 expression, with 25% of cases placed into different clinically relevant categories of PD‐L1 expression. PD‐L1 expression by immunochemistry is an exemplar of the challenges of utilising a heterogeneously expressed protein‐based predictive biomarker. Regardless of sampling technique, a good quality biopsy or FNA is likely to give a statistically representative PD‐L1 expression, although expression ranges close to clinically relevant cut‐offs of 1% and 50% remain a source of potential discordance.

## Introduction

Predictive biomarkers are utilised in the lung cancer setting to identify patients who are amenable to a range of targeted treatments and immunotherapies [1]. The analysis of proteins via immunohistochemistry (IHC) can be utilised alongside next‐generation sequencing and other techniques to provide comprehensive molecular profiling for predicting the response to a range of targeted treatments and immunotherapeutics [2].

PD‐L1 expression via IHC remains the only clinically validated predictive biomarker for response to PD‐1/PD‐L1 immune checkpoint blockade in the non‐small cell lung carcinoma (NSCLC) setting and is increasingly utilised in a range of other solid tumours [3, 4, 5, 6, 7].

Challenges of this powerful, but fragile, biomarker have been addressed with a range of novel solutions such as machine learning‐derived image analysis in assisting interpretation [2]. Prediction with PD‐L1 IHC remains imperfect, however, and challenges of pre‐analytical fixation, differences in sampling techniques, and tumour heterogeneity persist. These remain a practical challenge to achieving high accuracy and attaining the best predictive power [3, 8, 9, 10].

This study aimed to evaluate how pre‐analytical variables, including sampling modality and fixative type, impact the expression of PD‐L1 in NSCLC specimens with matched cytological fine‐needle aspirates (FNAs), core biopsies, and surgical specimens.

## Materials and methods

### Patient and specimen details

Patients with a pre‐surgical histologically confirmed diagnosis of NSCLC undergoing surgical resection met the inclusion criteria for this study. Exclusion criteria were as follows: patients undergoing neo‐adjuvant therapy; tumours that on the surgical specimen resection were found to be extra‐thoracic (metastatic) lung tumours; high‐grade neuroendocrine carcinomas, (e.g., small cell carcinoma); or any with limited quantity, defined as being stage T1a (<10 mm in size).

Tumours were classified according to the current World Health Organisation 5th edition (2021) [11] and staged as per the then current edition of the Union for International Cancer Control (UICC) TNM 8 staging system [12]. Accompanying clinical data were available from case‐note review. Ethical approval for the Liverpool Lung Project was granted by the Liverpool Research Ethics Committee (reference number 97/141).

### Specimen handling

Immediately following surgical resection, the lung or lung lobe was transported unfixed on ice directly to the pathology laboratory. Cold ischaemia time was not specifically recorded but was estimated to be between 1 and 2 h for each specimen. Upon receipt, each specimen was sampled to produce two FNA samples, two core biopsies, and two whole blocks of tumour tissue. The fresh specimen was assessed for suitability and tumour location and then incised via the pleura to demonstrate the tumour. Under direct vision, two FNA samples were taken using a syringe and 21‐gauge needle, with 2–3 passes taken for each sample. One FNA sample was then placed into an alcohol‐based fixative (Cytolyt™, Hologic, Manchester, UK) and the other was placed into formalin. Core‐needle biopsies were taken utilising a 19‐gauge automated cutting needle, with one core of tumour placed into formalin and one placed into the alcohol‐based fixative. Finally, two whole tumour blocks (approximately 15 × 10 mm each) were taken, with one placed into the alcohol‐based fixative and one into formalin.

The remaining specimen was then placed in formalin and retained for routine clinical processing as per local protocol.

### Fixative agents

Fixation used two agents. The first was formalin (10% neutral buffered formalin) that is standard of care for histology specimens. The alcohol‐based fixative was Cytolyt, a methanol‐based buffered solution that is free of formalin. Previous studies addressing alcohol‐based fixation have found impact is minimised if using a post‐alcohol formalin fixation step and is considered by expert consensus panels to be suitable for use when wishing to assess PD‐L1 expression [9].

### Sample preparation

The FNA specimens were centrifuged for 10 min at 2500 rpm, and the supernatant was decanted. After decanting the supernatant from all specimens after a second spin, agar was placed onto the pellet to produce a solid cell block. Pellets were then bio‐wrapped, placed into a labelled histology cassette, and processed overnight (12 h) in a Leica Peloris III HistoCore processor. FNA specimens had a post‐fixation step in 10% formalin for a minimum of 45 min while they were in the processor. The specimens were then embedded in paraffin wax. The core biopsies and whole‐block specimens were placed into standard tissue cassettes and embedded in paraffin wax following adequate fixation (12–24 h).

In such fashion, a total of six blocks were produced from each specimen.

### Detection and assessment of PD‐L1 expression

Tissue sections 4 μm thick from each block were stained with haematoxylin and eosin (H&E) to confirm the presence of tumour, appropriate diagnosis, and an assessment of tumour cell content and cellular quality. Specimens with fewer than 100 viable tumour cells were deemed inadequate and excluded in accordance with PD‐L1 assessment guidelines [13]. A serial section was stained for PD‐L1 utilising the Ventana SP263 antibody clone with a validated kit and protocol. Slides were scanned at ×20 magnification on the Roche DP200 digital slide scanner and viewed on the opensource QuPath software package [14].

Expression of PD‐L1 was assessed according to the Roche Ventana SP263 interpretation guide by two pathologists (AH and JRG) trained and experienced in its interpretation. Both pathologists were blinded to fixative modality. Each case was scored together on double‐headed microscopes, and a concordant TPS was agreed in all cases. The number of PD‐L1 positive tumour cells as a proportion of the total number of tumour cells (the tumour proportion score, TPS) was expressed as a percentage. Cases were also placed into the clinically relevant grouping of <1%, 1–49%, and ≥50% as well as a binary delineation of <50% and ≥50%.

### Statistics

Statistical analysis was performed using IBM SPSS statistics software, version 26 (IBM Corp). As all data were non‐normally distributed and paired, they were compared via Friedman test for three or more groups and Wilcoxon matched‐pair signed‐rank test for two groups. All *p* values are taken as significant if <0.05.

## Results

### Patients and specimens

A total of 62 patients were consented. Three patients had a final diagnosis of metastatic disease, and two had small cell carcinoma and were excluded from the study, leaving 57 patients. Patient and specimen details are summarised in Table 1: 34 adenocarcinomas, 22 squamous cell carcinomas, and 1 adenosquamous carcinoma were included. Two whole‐block, two core biopsy, and two FNA samples were produced from each specimen to produce 114 for each specimen type and a total of 684 specimens.

**Table 1:** Demographic and histological characteristics of the included patients and tumours

Characteristics	*N* (%)
Total number	57 (100)
Morphology	
ADC	34 (60)
SCC	22 (39)
ADSC	1 (1)
Median age (at diagnosis)	70 (range 53–83)
Gender	
Male	26 (46)
Female	31 (54)
Stage (at diagnosis)	
T1*	33 (58)
T2^†^	15 (26)
T3	6 (11)
T4	3 (5)
Nodal stage (at diagnosis)	
N0	45 (79)
N1	5 (9)
N2	7 (12)

* Includes T1b–c.

^†^ Includes T2a–b.

**Figure 1:**
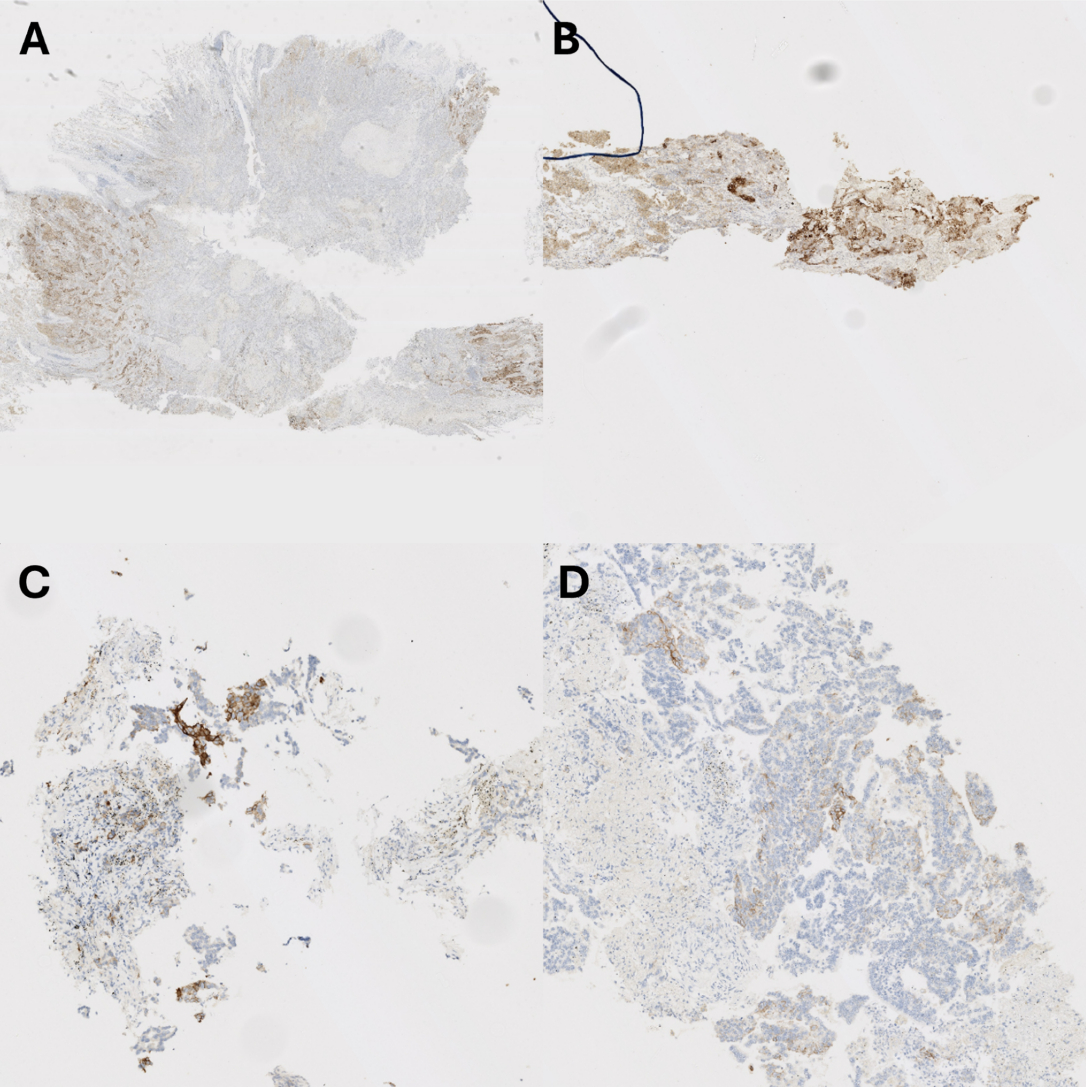
(A) Whole‐block (alcohol fixed), (B) core biopsy (alcohol fixed), (C) FNA (formalin fixed), (D) FNA (alcohol fixed). Matched NSCLC specimens stained for PD‐L1 (SP263 clone). Note the striking heterogeneity of the whole‐block specimen. Focal sampling of this via biopsy or FNA can result in a PD‐L1 TPS that is notably higher or lower. While there is reduced staining in (D), it is difficult to ascribe this to purely artefact or heterogeneity, although the presence of positive internal controls suggests heterogeneity is the likely cause.

**Figure 2:**
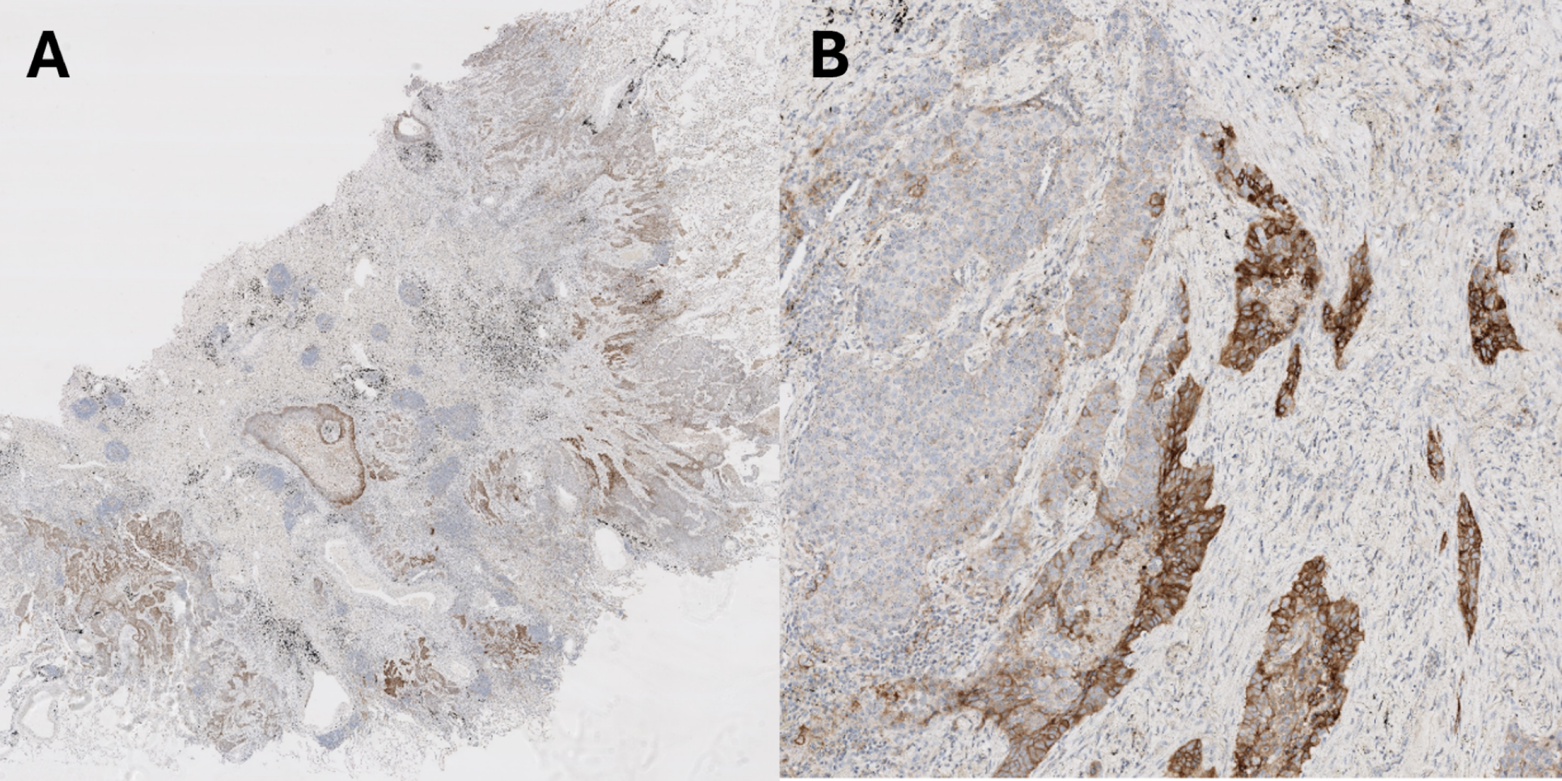
(A) Whole‐block (formalin fixed), (B) higher power view. Striking heterogeneity with sharply delineated areas.

**Figure 3:**
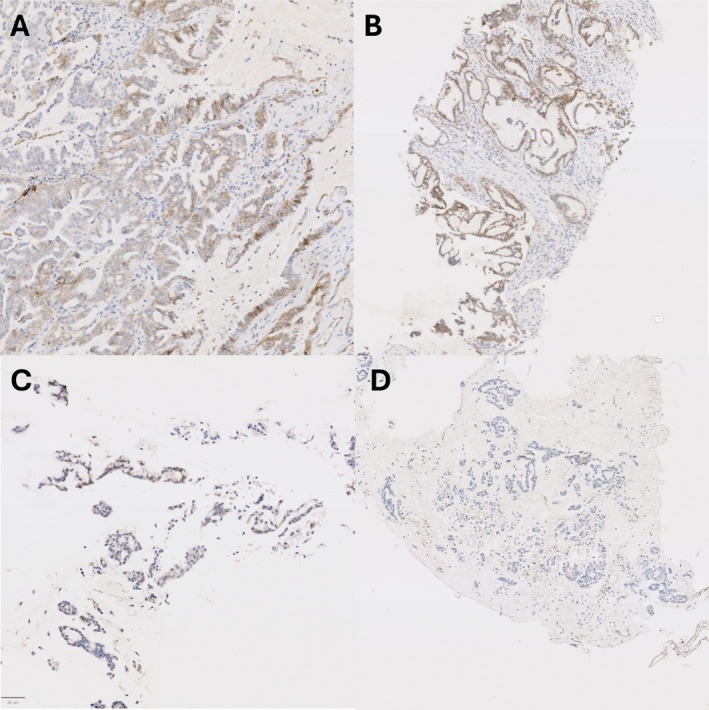
(A) Whole‐block (alcohol fixed), (B) core biopsy (alcohol fixed), (C) FNA (formalin fixed), (D) FNA (alcohol fixed). Matched NSCLC specimens stained for PD‐L1 (SP263 clone). Strong PD‐L1 expression is seen in (A–C) but with notable negative staining on the alcohol fixed FNA (D). In contrast to Figure 1, with a lack of positive internal controls, this is likely artefactual in nature.

Examples of PD‐L1 staining in matched samples are given in Figures 1, 2, 3, including cases of marked heterogeneity (Figures 1 and 2) and staining differences between fixatives (Figure 3).

### Adequacy and quality of specimens

All 114 whole‐block specimens had adequate whole‐block sampling for both fixative agents. Of the 114 core biopsies, 9 (8%) had insufficient tumour cells (4 fixed in alcohol and 5 fixed in formalin). Of the 114 FNA samples, 25 (22%) had insufficient tumour cells (12 fixed in alcohol and 13 fixed in formalin). There was no significant difference in adequacy rates between fixative agents. There was no appreciable difference in the quality of cellular morphology between the alcohol‐fixed and formalin‐fixed tissue with all adequate cases considered suitable for analysis.

### Comparison of PD‐L1 expression between fixative agents as a continuous variable

There was no significant difference in average TPS between fixative agents for both whole‐block specimens (alcohol versus formalin: 19 versus 18, *p* = 0.346) and core biopsies (alcohol versus formalin: 19 versus 18, *p* = 0.448). FNA specimens fixed in alcohol had a significantly reduced average TPS compared to those fixed in formalin (7 versus 19, <0.001). FNAs fixed in alcohol also had a significantly lower TPS than alcohol‐fixed core biopsies and surgical blocks (19/18 versus 7, *p* < 0.001). When excluding the alcohol‐fixed FNAs, there was no significant difference in average TPS between sampling modalities.

### Comparison of PD‐L1 expression between fixatives as clinically relevant categorical groups

PD‐L1 TPS for each specimen as a clinical grouping is summarised in Table 2.

**Table 2:** PD‐L1 TPS provided as clinically relevant categories for each method of sampling and fixative agent

	Resection alcohol	Resection formalin	Core Bx alcohol	Core Bx formalin	FNA alcohol	FNA formalin
<1%	18	25	23	24	30	18
1–49%	28	23	20	19	13	18
≥50%	11	9	10	10	2	8
Inadequate	0	0	4	5	12	13

Bx, biopsy; FNA, fine‐needle aspirate; PD‐L1, programmed death‐ligand 1; TPS, tumour proportion score.

When comparing matched whole blocks between fixative agents, 10 cases (18%) were placed into different clinical groups. There was no significant difference in the classification of tumours when utilising the 50% cut‐off (four cases, *z* = −1.414, *p* = 0.157).

When comparing matched core biopsies, seven (12%) were placed into different clinical groups. There was no significant difference in the classification of tumours when utilising the 50% cut‐off (four cases, *z* = −1.414, *p* = 0.157).

When comparing matched FNAs, 15 (26%) cases were placed into different clinical groups, all of which were alcohol‐fixed specimens placed into lower groups. All five (9%) FNAs placed into a lower group utilising the 50% cut‐off were alcohol‐fixed, which was a significant reduction (*z* = −2.646, *p* = 0.008).

### Comparison between PD‐L1 expression between sampling methodologies

There was no significant difference in average TPS between formalin‐fixed tissue sampled as a whole‐block, core biopsy, or FNA (18 versus 18 versus 19, *p* = 0.791). There was also no difference when comparing alcohol fixed tissue sampled as a whole‐block or core biopsy (19 versus 19, *p* = 0.491) but a significant reduction when comparing alcohol‐fixed FNAs (7 versus 19 versus 19, *p* < 0.001).

When considering clinical groupings for formalin‐fixed tissue sampled as a whole‐block, core biopsy, or FNA, 13 (23%) cases were placed into different groups, and for alcohol‐fixed tissue specimens, 20 (35%) were placed into different groups, largely as a result of alcohol‐fixed FNAs returning a lower TPS.

There was no significant difference in the classification of tumours when utilising the 50% cut‐off in formalin‐fixed tissue [three (5%) of the core biopsies and five (9%) of the FNAs were placed into different groups compared to their matched whole‐section specimen, *Χ* ^2^ = 3, *p* = 0.223]. When using a 50% cut‐off for alcohol‐fixed tissues, there was no significant difference between whole‐section specimens and core biopsies (*z* = 1, *p* = 0.317) but a significant reduction for the alcohol‐fixed FNAs (*Χ* ^2^ = 16.2, *p* < 0.001).

## Discussion

Predictive biomarkers for the use of targeted treatments and immunotherapies have revolutionised the management of patients with NSCLC and changed the way pathology services have been delivered [1, 10]. While much focus is on nucleic acid analysis, and more latterly the role of circulating biomarkers, it is important not to neglect the value of proteins in tissue that can provide comprehensive data as to the nature of the tumour immune microenvironment and powerful predictive biomarkers. PD‐L1 expression as a biomarker for immunotherapy is established in a wide range of solid tumours and serves as the exemplar for protein‐based biomarkers such as for other immune checkpoint inhibitors and antibody drug conjugates [2].

The study of proteins in the routine clinical pathology laboratory is largely dependent on immunochemistry [15]. This technology has proven itself to be robust, relatively cheap, and easy to implement, with PD‐L1 expression used globally to predict response to immunotherapy [1]. It does, however, have a number of limitations; it is generally used as a single‐plex technique, is prone to error resulting from pre‐analytical conditions, can be impacted by antigen masking and differential retrieval, may have multiple non‐identical assays, and is hampered by the challenges of assessment and inter‐observer discordance.

This paper builds on previous work in addressing perhaps the major outstanding challenge when assessing PD‐L1 expression and predictive protein‐based biomarkers in general: tumour heterogeneity. The classification of patients into those whose tumours have less than or greater than 50% PD‐L1 TPS has profound implications on patient management, and tumour heterogeneity and sampling error can potentially incorrectly render patients ineligible for immunotherapies if misclassified as less than 50% [2].

In our study, when focusing on sampling technique alone, there is no statistically different change in mean PD‐L1 TPS between whole tissue sections, core biopsies, or FNAs, suggesting that each technique will, broadly speaking, return a representative value. Nonetheless, it is interesting to observe that, when comparing matched two‐section blocks, while no significant difference is observed in average PD‐L1 expression, there are several instances when one block shows a clinically relevant change in PD‐L1 expression, with several cases showing no PD‐L1 expression in one block but 1–2% in another. Fixation does not seem to be the reason for this in the whole‐block mounts: there is no change in average TPS or the number of viable cases between the fixative agents, and the alcohol‐fixed whole‐blocks actually showed fewer negative cases. Rather, it appears to be a result of genuine PD‐L1 expression heterogeneity. Heterogeneity can be scatter‐gun in its expression but can also be extremely focal. Examples of very focal PD‐L1 expression are shown in Figures 1 and 2; and it is from such cases that considerable individual variation between sampling techniques has been observed. Evidently, it appears that focal PD‐L1 expression can be appreciated on large surgical specimens, with sampling via biopsy or FNA prone to over‐ or under‐calling the TPS depending on whether they hit or miss the focally expressing area of tumour.

As with previous studies on the subject, it seems one to two blocks from surgical sections will provide a reliable illustration of PD‐L1 expression in most cases [8, 16], but for non‐surgical sampling this remains an area of potential error for which no obvious solution is currently identified [17, 18, 19, 20].

The other significant finding in this study is that of a statistically and clinically relevant reduction in PD‐L1 expression in alcohol‐fixed FNA samples (example shown in Figure 3). This is a surprising observation to us, as previous studies have illustrated the robust nature of FNA samples for PD‐L1 expression, particularly when utilised as formalin‐fixed cell blocks [9, 18, 21]. This is most likely explained by the delay to formalin fixation in the affected cases; most of the impacted cases were from very late 2019 and early 2020 when the enforced closing of research laboratories due to COVID‐19 resulted in delayed processing of these specimens. It is interesting to note that the same reduction in PD‐L1 expression is not seen in either the core biopsy or whole‐block specimens, suggesting the change is not necessarily due to the alcohol fixation *per se*, but rather the inherent nature of dispersed groups of cells from FNAs that are less physically robust and more prone to artefactual change, whereas intact tissue is more resistant [21]. It is hoped that, as demonstrated in previous studies and in concordance with guidelines, rigid adherence to limited time within alcohol‐based fixatives will not yield false negative results [22].

There are several limitations in this study. First is the question of how best to compare PD‐L1 data. Comparing PD‐L1 TPS can be done mathematically or clinically. While data as a continuous variable provides an objective outcome, relatively large changes in PD‐L1 may have no impact on clinical management (e.g., the difference between 55% and 100%) but relatively small changes in PD‐L1 at key cut‐off points may dramatically alter patient management (e.g., 49% versus 51%) [23, 24]. This study has therefore looked at PD‐L1 as both a continuous variable and as categorical data, while acknowledging the inherent limitations of both.

Inter‐observer variation is also a potential confounding factor in scoring PD‐L1 [18, 24, 25]. We feel that the type of specimens involved warranted an open and free discussion throughout, but we acknowledge that a lack of initial blinded independent observations is a potential confounding factor. Delayed time to formalin fixation, as described above, is also a potential confounder. Despite a consistent approach to sampling, we did not specifically record cold ischaemia time, a pre‐analytical factor known to potentially reduce antigenicity in IHC assays [18]. We also did not use a separate protocol for PD‐L1 immunochemistry for alcohol‐fixed specimens compared to formalin‐fixed specimens. While previous studies have shown the validity of this approach [9] it is possible that a dedicated protocol may address some of the apparent loss of PD‐L1 expression in alcohol‐fixed FNAs.

The use of PD‐L1 TPS to guide patient management remains a key aspect of lung pathology but is hampered by several pre‐analytical and biological factors. Sampling error from tumour heterogeneity is a challenge, as is the fact that a significant proportion of patients with NSCLC will have only cytological specimens for predictive profiling [26]. We found that all sampling modalities, including cytological specimens, generally return a comparable and representative PD‐L1 TPS result. Formalin fixation was appropriate for all specimen types. Our unexpected finding was that alcohol‐fixed FNAs were inferior for scoring PD‐L1 in contrast to other studies, but we hypothesise this is likely due to delayed time to a post‐alcohol formalin fixation step.

## Conclusions

It has been well documented that tumour heterogeneity at a nucleic acid and protein level exists across space and time, with changes also seen following treatment [8, 17, 27, 28, 29, 30]. What is apparent is that the results are consistently inconsistent; heterogeneity is neither easily explained nor easily predicted. Indeed, some studies have suggested that differences in sites may predict response to immunotherapy variably [25, 27, 30, 31].

This study adds to the body of evidence that, if biopsy, cytological, and surgical specimens are handled appropriately, they can all be used to yield a valid PD‐L1 TPS for PD‐L1 expression in NSCLC to guide clinical management. Careful handling of specimens is critical to ensuring an accurate immunochemical stain; in particular, FNAs appear to be susceptible to false under‐scoring if handled poorly. Tumour heterogeneity remains a fascinating but incompletely understood phenomenon and must remain a consideration for current and future protein‐based biomarkers when used to guide targeted treatments and immunotherapies.

## Author contributions statement

AH and JRG designed the study. MS performed the operations and oversaw patient consent. AH, MPD, NK and JKF supervised the collection of specimens. AH and JRG characterised the specimens. AH and NK prepared the tissue for analysis. NK performed immunohistochemical analysis and digital slide scanning. AH and JRG interpreted the data. AH analysed the data. AH wrote the paper. All authors critically reviewed the paper.

## Data Availability

The data are available on request from the authors.
